# Influence of Individual Cognition, Satisfaction, and the Theory of Planned Behavior on Tenant Loyalty

**DOI:** 10.3389/fpsyg.2022.882490

**Published:** 2022-07-20

**Authors:** Chun-chang Lee, Wen-chih Yeh, Hung-chung Chang, Zheng Yu, Zheng-yang Tsai

**Affiliations:** ^1^Department of Real Estate Management, National Pingtung University, Pingtung City, Taiwan; ^2^Department of Real Estate Management, HungKuo Delin University of Technology, New Taipei City, Taiwan; ^3^Department of Business Administration, Chihlee University of Technology, New Taipei City, Taiwan; ^4^Department of Land Economics, National Chengchi University, Taipei City, Taiwan

**Keywords:** cognition, affection, conation, theory of planned behavior, situational influences, tenant satisfaction, tenant loyalty

## Abstract

This study proposed a conceptual framework and explored the influence of personal-level variables such as cognition, affection, conation, and attitude, as well as the theory of planned behavior, on tenant loyalty using situational influence as a moderator variable. Structural equation modeling was employed for parameter estimation. A total of 315 questionnaires were administered to housing tenants residing in Kaohsiung City, of which 300 were recovered, a recovery rate of 95.2%. The results are as follows: Tenants’ cognition, affection, and conation positively influenced their attitude toward their tenancy; tenants’ attitude significantly and positively influenced their satisfaction and loyalty; tenant satisfaction significantly and positively influenced tenant loyalty; perceived behavioral control significantly and positively influenced tenant loyalty; and subjective norms significantly and positively influenced tenant loyalty. Regarding the influence of attitude on tenant loyalty, the inclusion of a situational influence-based moderator variable—i.e., whether a tenant has friends or relatives living nearby—revealed that the presence of this variable significantly diminishes the influence of attitude on loyalty. The findings indicate that landlords could improve the quality of their services by implementing sustainable management measures to strengthen tenant satisfaction and, consequently, tenant loyalty.

## Introduction

Renting a house is a process most people experience. According to data from the 2018 Population and Housing Census conducted by the Directorate General of Budget, Accounting and Statistics (2018), Executive Yuan, approximately 876,009 people in Taiwan rent a house. There are approximately 86,659 tenants in Kaohsiung City, which accounts for 9.8% of the tenant population in Taiwan, making the city among the top three cities/counties nationwide in terms of tenant population. This is a testament to the importance of the rental market in Kaohsiung City. In addition, it is even more important for landlords to gain a competitive advantage by providing superior services to ensure their longevity in the market as it matures.

The theory of planned behavior (TPB) can be used to analyze the factors affecting tenant loyalty. Ajzen and Martin (1980) suggested that a person performs a particular behavior because of the influence of behavioral intention. Ajzen (1991) identified intention as a salient predictor of actual human behaviors. The TPB is rooted in the theory of reasoned action and its current form was adapted by Ajzen (1991), whereby behavioral beliefs, normative beliefs, and control beliefs were, respectively, included into the components of attitude, subjective norms, and perceived behavioral control (PCB). The TPB is unique because it not only measures human behavior through the predictor variables of attitude and subjective norms but also considers the factor of PCB. In other words, the TPB predicts that when a person displays a more positive attitude toward a particular behavior and when they perceive a greater level of normative pressure from their surroundings, they will perceive greater control over the said behavior and, ultimately, will have a stronger intention to perform that behavior. In recent years, the TPB has been used to study transactional behaviors. The literature has shown that it has a significant explanatory power for a person’s intention to purchase different types of products (Chiu et al., 2014). To gain a more in-depth understanding of how behavioral intentions are strengthened, one should begin with the factors of attitude, subjective norms, and PCB. Ajzen (1991) noted that cognition, affection, and conation are the antecedents of attitude. In early studies on this subject matter, Greenwald (1968) proposed that when a person receives new information, they attempt to link the information with their previously acquired knowledge, affection, or attitude, and their previous perceptions may be altered during the linking process. In other words, a tenant will inspect their own experiences in the past or refer to the experiences of other tenants and change their existing attitude and, subsequently, their tenant loyalty. Indeed, the attitudes of tenants toward their tenancy are influenced by cognition, affection, and conation.

Furthermore, discussions on the behavioral intentions of tenants should consider the relationships between tenants’ attitude, satisfaction, and loyalty. Djebarni and Al-Abed (2000) highlighted that tenant satisfaction is the satisfaction of an individual or family toward the condition of their rented house. It is also an indicator of housing satisfaction as well as a component in the continuum of overall satisfaction. Tenant satisfaction is a complex attitude that cannot be measured by proposing a simple question. Scholars generally agree that attitude affects satisfaction whereas satisfaction affects loyalty. Jiboye (2008) concluded that the cognitive imaged shaped by tenants through the process of perception shapes the basis of their personal attitude and feelings toward each component, and the totality of these attitudes and feelings forms the basis of tenants’ satisfaction with their house.

Interestingly, situational influences may interfere with or moderate the relationship between tenant attitude and tenant loyalty. Situational influences are factors that arise from the factors considered by a customer, as well as the time and place, which subsequently affects the customer’s decision-making process. They can affect customer behaviors at any stage during the purchasing process, and may also shorten, prolong, or halt the purchasing process (Pride et al., 2008). Ajzen (1991) found that against the backdrop of different behaviors or situations, attitudes, subjective norms, and perceived behavioral control have varying degrees of influence on intentions. Assael (1995) asserted that a customer makes a purchase only when the customer, situation, and product affect one another. Thus, we sought to examine whether the influence of tenant attitude on tenant loyalty is moderated by situational influences.

In conclusion, it’s found, by referring to the previous discussion on relevant issues, that the Theory of Planned Behavior (TPB) is mainly applied to the behavioral intention of word-of-mouth on tourists’ purchase of souvenirs (Lee, 2020) and the model testing of college students’ cheating behavior (Koc and Memduhoglu, 2020); and that the situational influence is widely used in investigating the strategies of household consumption behavior (Kuhe and Bisu, 2019), integration of social application software into aesthetic education (Chen et al., 2020), etc.; and that attitude, subjective norms, and perceived behavioral control (PCB) are mainly used to study the behavioral intentions and habits of enterprise employees in energy saving (Chen and Chen, 2019), and those of young Vietnamese consumers in purchasing foreign coffee (Su and Kang, 2019). They are rarely used in the analysis of the housing rental industry. This study investigated the loyalty of tenants in Kaohsiung City. The TPB was used as a basis for analysis in which three variables of personal perception—cognition, affection, and conation—were set as antecedents of attitude. Tenant satisfaction served as a bridge between the attitude-loyalty relationship, and situational influences were set as a moderator variable between attitude and tenant loyalty. A conceptual model was developed to analyze the relationship between tenants and landlords, in which parameter estimation was performed *via* structural equation modeling (SEM). Customer support is a key factor that determines a company’s sustainability. Customers who feel satisfied with a product or service are more likely to become loyal customers and may actively share relevant information about the product or service with others through word-of-mouth. This effect is essential for ensuring the sustainability and success of an actor in the real estate industry against temporal changes and rising competition.

The main contribution of this study is that, based on the traditional TPB model, personal perception variables including cognitive, affective and conative elements were taken into account, and the moderating effect of situational influence was also considered for an empirical study on tenant loyalty. This study not only further expanded the TPB model in theory by including personal perception variables and situational influence into the model, making the TPB model more complete and breaking through in theory, but also made the expanded TPB model more consistent with the actual situation of tenancy, thus making the empirical results of this study more representative.

## Literature Review and Research Hypotheses

Rosenberg and Hanland (1960) proposed the cognitive, affective, and conative components of attitudes. Petty and Wegener (1999) emphasized the important role cognition holds in the process of attitude formation. Repeated exposure to an attitude object allows an individual to strengthen their confidence by having more opportunities to process more information, reassert their attitudes, and increase their perception toward a brand. Howard (1994) argued that when customers make purchase decisions, their perception, confidence, and attitudes are affected by the information about a product whereas their confidence and attitudes are also affected by their perceptions, which subsequently affects their behavioral intention. The extent of their behavioral intention further determines their actual purchase behaviors. Thomas et al. (2019) investigated the Indian enterprises in terms of social network, organizational learning and organizational cognition by taking the attitude as moderating effect. The research results showed that, according to the Multiple Regression Analysis (MRA), social network is strongly associated with the organizational learning, social network is interactive with the organization cognition, and the organizational cognition has positive effects on the attitude.

Allen et al. (1992) proposed that affection predicts behavior better than cognitive assessment for two reasons: when previous behaviors are regarded as legitimate, the development of cognitive appraisal will be inhibited, and recollection of emotion may be a determinant of behavior; once a behavior becomes a habit in which past experiences are repeated, the behavior is relatively devoid of cognitive assessment and, in a specific context, may be guided by previous emotional experiences. Bennett et al. (2005) argued that affection is an attitudinal expression in commercial purchase behaviors because it frequently balances the expression of attitudes and shapes a customer’s brand loyalty toward a product. In a case study of Kinmen County, Taiwan carried out by Chen (2020), the residents on the island are investigated with respect to their affective attachment to the island, well-being and attitude toward tourism development. According to the research, the island’s residents attachment to and recognition of where they live arising from their affective attachment to the residence directly influence their attitude toward tourism development. Therefore, the affection has a significant positive influence on attitude.

Park et al. (2008) asserted that customers’ emotions could affect their intentions to purchase a product. As shown in a study on the attitude toward scientific research carried out by Ünver et al. (2018) among 375 nursing graduates in a university of Turkey, students are not only more engaged in the scientific activities but also can improve their attitude toward scientific research if provided student grants as appropriate (conation). From this, we can see that conation has positive effects on attitude. Previous studies have regarded cognition, affection and conation as a whole, making it difficult to find the individuality of each. In this study, these three aspects were separately investigated for detailed research and discussion, with the housing rental industry that was scarcely investigated was studied in the hope of new research results and analysis. H1 to H3, as follows:

**H1:** Cognition has a positive and significant influence on attitude.

**H2:** Affection has a positive and significant influence on attitude.

**H3:** Conation has a positive and significant influence on attitude.

Tenant satisfaction is a multidimensional cognitive attitude whereby its expression cannot be measured by proposing a simple question. The cognitive image shaped by tenants through the process of perception shapes the basis of their personal attitude and feelings toward each component, and the totality of these attitudes and feelings forms the basis of tenants’ satisfaction with their house (Jiboye, 2008). By means of the attitudinal process of perception, when a customer has a good impression of a certain product, they will become more satisfied with it (Jiboye, 2008). Chen and Shiu (2016) analyzed customer relations management and tenant satisfaction based on tenants’ occupation (students and workers) and found that tenants have higher satisfaction when they perceive a better quality of service, especially when they have a good attitude in handling issues. The more satisfied tenants are, the more willing they are to renew their tenancy. Dimitriades (2006) stated that loyalty is a customer’s positive attitude whereby they identify with a company and subsequently make repeat purchases, recommend the company to others, and thereby affect others’ purchase behaviors. When robust affective experience or satisfaction becomes the basis of a customer’s strong relative attitudes toward an offer, they may gravitate toward spreading a positive word-of-mouth in addition to repeat sponsoring (Westbrook, 1987). In a study on cognition-affection-cognitive model of a fashion brand conducted among 300 American graduates by Han and Choi (2019) with the application of SEM (structural equation modeling), it’s found that consumers who know more about fashion brands have stronger affective attachment to brand, in which such affective attachment will directly affect their attitude toward the brand, so that they will build the loyalty to the brand for repurchase. The biggest difference between housing rental and general commodities lies in its long-term and regional restrictions, which are all affected by the attitude of the tenant. On this basis, we propose H4 and H5 as follows:

**H4:** Attitude has a positive and significant influence on tenant satisfaction.

**H5:** Attitude has a positive and significant influence on tenant loyalty.

Barnes (2006) asserted a close interdependence between customer satisfaction and customer loyalty. Satisfaction positively affects customer loyalty; tenants who receive services of excellent quality will feel more satisfied, and this high level of satisfaction will positively affect their loyalty (Calik and Balta, 2006). Fuentes-Blasco et al. (2014) agreed that satisfaction has an active and salient influence on customer loyalty. Customer satisfaction also positively affects repurchase behaviors, which is an expression of customer loyalty. Lee et al. (2021) conducted a study on the loyalty of the tenants by applying SEM for analysis. As shown in the study, practically and technically speaking, the tenant satisfaction has a mediating effect on the tenant satisfaction. As such, tenant satisfaction has a significant positive effect on the tenant loyalty. Therefore, we propose H6 as follows:

**H6:** Tenant satisfaction has a positive and significant influence on tenant loyalty.

Ajzen (1991) stated that perceived behavioral control is a person’s perceived level of difficulty when performing a certain behavior. A customer with a stronger perceived behavioral control will have stronger purchase intentions. According to Gasiorowska (2014), in product buying and selling, customers are affected by the perceived behavioral control of whether they are able to afford a product. As affordability is a variable that affects purchase intentions, perceived behavioral control will effectively affect purchase intentions. As shown in a TPB-based analysis carried out by Lee et al. (2021) among tenants in Kaohsiung City, Taiwan, perceived behavioral control (PCB) has a significant positive effect on the tenant loyalty, that is, individual desire and demand have a relative relationship with loyalty, the higher the perceived behavioral control, the higher the tenant loyalty. It is worth noting that external factors, such as the COVID-19 pandemic, can have an impact on a person’s anticipation and behavior control (Birtus and Lăzăroiu, 2021; Rydell and Kucera, 2021; Watson and Popescu, 2021). Therefore, we propose H7 as follows:

**H7:** Perceived behavioral control has a positive and significant influence on tenant loyalty.

Berkowitz (2004) found that misperception occurs when a person overestimates or underestimates the benefits of the attitudes or behaviors of/in a group. People can misguide their social groups or larger communities through various means, thus affecting the behaviors of others. Ajzen and Martin (1980) proposed that intention comprises subjective norms, which refer to a person’s belief that significant others should or should not perform a particular behavior as well as their motivation to comply with these views. Chen et al. (2019) conducted a study on behavioral intention and loyalty based on the subjective norm and perceived behavioral control of college students’ use of public bicycles. The study showed that personal subjective norm will affect perceived behavioral control and have a significant positive impact on the use intention and loyalty. Based on this reasoning, tenants are affected by subjective norms when society displays a good attitude or appraisal toward the tenancy, thus affecting their willingness to renew their tenancy. Against this backdrop, we propose H8 as follows:

**H8:** Subjective norms have a positive and significant influence on tenant loyalty.

Wells and Prensky (1996) argued that situational influences are a notable factor that affects purchasing activities in which customers change their original purchase decisions. Situational factors can affect customer behaviors at any stage during the purchasing process, and may also shorten, prolong, or halt the purchasing process (Pride et al., 2008). Blackwell et al. (2005) proposed that personal factors and situational factors should be considered when explaining customer choices. Ajzen (1991) highlighted that against the backdrop of different behaviors or situations, attitudes, subjective norms, and perceived behavioral control have varying degrees of influence on intentions. Assael (1995) asserted that a customer makes a purchase only when the customer, situation, and product affect one another. Customers are easily stimulated when they perceive others as having a superior product, which prompts them to be attracted toward a product and hence develop benign envy, which further results in impulsive purchase behaviors (Crusius and Mussweiler, 2012). Chao and Yu (2018) conducted a survey on the trend of international mathematics and science education achievement of 5,042 students in 2011 to verify the social cognitive career theory (SCCT). In addition, school, teachers and parents’ attitudes were taken as the variables of situational influence. The study showed that only teachers’ influence is moderately important, while school and parents’ influence is relatively small. The high housing rental cost in Taiwan determines that relatives and friends’ attitudes and suggestions are important influence factors. As a result, the follow-up study will define situational influence variables as whether relatives and friends live near the rental premises. We propose H9 as follows:

**H9:** The influence of attitudes on tenant loyalty is moderated by the availability of friends and family living nearby.

## Methods

**FIGURE 1:**
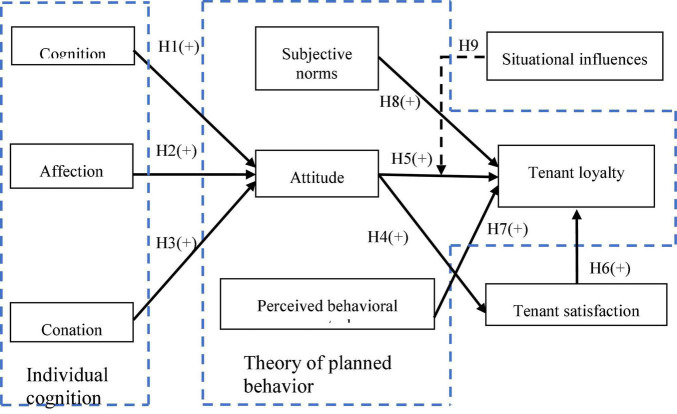
The conceptual framework.

We first developed a conceptual framework as shown in Figure 1, then defined the operational definitions of the variables and designed a questionnaire, followed by sampling and data collection.

### Conceptual Framework

The nine hypotheses proposed in this study were compiled into the conceptual framework, shown in Figure 1.

### Operational Definitions of Variables

The variables in this study consist of four personal variables (cognition, affection, conation, and attitude) and five other variables (subjective norms, situational influences, perceived behavioral control, tenant satisfaction, and tenant loyalty). The operational definitions of the variables are as follows:

#### Cognition

Bolton and Drew (1991) developed a model for assessing service quality and value, in which perceived service, expected service, and uncertainty serve as the functions of service quality. Furthermore, according to Erevelles et al. (1999), customers’ perceptions of quality are influenced by the perceived risk level. We adopted Bolton and Drew’s (1991) concept of perceived service and Erevelles et al.’s (1999) concept of perceived risk as our operational definition of cognition.

#### Affection

Pugh (2001) argued that positive emotion, rather than negative emotion, can lead to higher customer satisfaction. In this regard, to ensure that every consumption experience generates positive value, strong affection, and feelings worth recollecting among customers, many organizations attach great importance to the verbal and non-verbal interactions between services providers and customers, amending intentions and supervising the etiquette displayed by employees to customers (Hochschild, 1983). We adopted Pugh’s (2001) concept as our operational definition of affection.

#### Conation

Merriam-Webster (1993) highlighted that conation is the innate motivation or striving toward a goal and the willingness to complete a task. Conation differs from cognition, which is defined as the mental processes involved with thinking, learning, and memory throughout the process of knowledge acquisition. We adopted Merriam-Webster’s (1993) concept as our operational definition of conation.

#### Attitude

Fishbein and Ajzen (1975) classified two types of attitude: (1) Attitude toward a behavior, which refers to the positive or negative assessment of the results of performing a behavior and includes practical assessment (conducive or not conducive), experiential assessment (enjoyable or not enjoyable), and overall assessment (good or poor); (2) Attitude toward an object, which refers to the attitudes toward a person, matter, object, or environment in addition to a behavior. We adopted Fishbein and Ajzen’s (1975) study on attitude as our operational definition of attitude toward a behavior and target object.

#### Tenant Satisfaction

Tenant satisfaction encompasses four different types of satisfaction: (1) Satisfaction with a house; (2) Satisfaction with the services provided, including maintenance; (2) Satisfaction with the rent paid, including the house and services; (4) Satisfaction with the residential area (Onibokun, 1974). Other studies have indicated that rental satisfaction can be measured through the attributes of a house, such as the function; physical livability; quantity, quality, and adequacy of social and community facilities; government policies and the traits and efficiency of government workers; ease of living; familial and environmental conditions and maintenance; maintenance of residential facilities; privacy; territory; and neighborhood security (Onibokun, 1974; Oladapo, 2006). We divided the operational definition of satisfaction into service contents, service pricing, service quality, and service complaints based on studies by Onibokun (1974); Satsangi and Kearns (1992), and Oladapo (2006).

#### Tenant Loyalty

Oliver (1997) defined loyalty as the willingness to repurchase the same product or service regardless of the cost while ignoring other options. Loyalty consists of attitudinal loyalty and behavioral loyalty; the former concerns consumers’ psychological level, whereas the latter concerns the actual purchase behaviors adopted by consumers. We divided the operational definition of loyalty into attitudinal loyalty and behavioral loyalty based on a study by Oliver (1997).

#### Perceived Behavioral Control

Ajzen (1991) posited that perceived behavioral control consist of two important constructs: self-efficacy, which concerns the internal psychological notion of an individual, and external resource constraints, which are the facilitating conditions that promote or hinder a behavior. In other words, it is the extent to which a person perceives the influence of these resources, opportunities, or barriers when they perform a behavior. Taylor and Todd (1995) decomposed perceived behavioral control into perceived convenience and self-efficacy. We divided the operational definition of perceived behavioral control into perceived convenience and self-efficacy based on studies by Ajzen (1991) and Taylor and Todd (1995).

#### Subjective Norms

Cialdini and Goldstein (2004) identified four constructs of subjective norms as follows: (1) injunctive norms: telling others what can or cannot be done in certain circumstances; (2) descriptive norms: conveying information related to the recurring behaviors of others in certain circumstances; (3) personal norms: a person’s awareness of what can or cannot be done throughout the process of growing up; (4) Norm of reciprocity: reciprocating the favors of others. We adopted Cialdini and Goldstein’s (2004) construct as our operational definition of subjective norms.

#### Situational Influences

Situational influences must consider various factors that affect a customer’s decision-making process, such as the factors that contribute to an event, as well as time and location constraints. Drossos et al. (2014) divided situational influences into situations in which a person plays an active role (such as product involvement, browsing, and time and cost constraints) and situations in which a person plays a passive role when exposed to various stimuli (such as the sudden urge to satisfy oneself mentally under the influence of price rebates, social influence, product characteristics, and sensory stimuli). Both situations may influence a person’s tendency to engage in impulse buying. Because the latter fits the research implications of this study, we adopted Drossos et al.’s (2014) passive component of situational influences as our operational definition.

### Questionnaire Design

The questionnaire comprised two sections: the first covered the participants’ basic information, including their gender, age, occupation, and length of tenancy; the second covered items regarding cognition, affection, conation, attitude, tenant satisfaction, tenant loyalty, perceived behavioral control, situational influences, and subjective norms. Based on studies by Bolton and Drew (1991); Erevelles et al. (1999), and Chen and Shiu (2016), cognition covered five items across two sub-constructs: perceived services (two items) and perceived risk (three items). Based on a study by Pugh(2001), affection covered three items. Based on studies by Merriam-Webster (1993) and Stylos et al. (2016), conation covered two items. Based on a study by Oliver (1997), attitude covered four items across two sub-constructs: attitude toward a behavior (three items) and attitude toward a target object (one item). Based on studies by Oliver (1997); Oladapo (2006), and Chen and Shiu (2016), tenant satisfaction covered six items across four sub-constructs: service content (two items), service pricing (one item), product quality (one item), and customer complaints (two items).

Based on studies by Oliver (1997); Hsiao et al. (2006), and Chen and Shiu (2016), tenant loyalty covered three items across two sub-constructs: attitudinal loyalty (two items) and behavioral loyalty (one item). Based on studies by Ajzen (1991); Taylor and Todd (1995), and Liu and Wu (2007), perceived behavioral control covered four items across two sub-constructs: perceived self-efficacy (three items) and perceived convenience (one item). Based on a study by Cialdini and Goldstein (2004), subjective norms covered three items across three sub-constructs: injunctive norms, personal norms, and norm of reciprocity (one item for each sub-construct). Based on a study by Hawkins and Coney (2003), situational influences covered one item. All items were measured on a five-point Likert scale, with responses varying by the level of agreement from strongly disagree, disagree, neutral, agree to strongly agree. The questionnaire items are shown in Table 1.

**TABLE 1:** Questionnaire items and references.

Measurement construct	Item		References
(1) Cognition			
Cognition	Perceived service	1. To me, a landlord should provide professional and precise services	Bolton and Drew, 1991; Chen and Shiu, 2016
		2. To me, a landlord should provide trustworthy services.	
	Perceived risk	1. I think the area around the house I am currently renting is safe and secure.	Erevelles et al., 1999; Chen and Shiu, 2016
		2. I think the area around the house I am currently renting has excellent public facilities.	
		3. I think the house I am currently renting has a good fire safety system.	
(2) Affection			
Affection	1. I am gladly willing to live in the house I am currently renting.		Pugh, 2001
	2. I like to discuss the house I am currently renting with other people.		
	3. I have an affective bond with the house I am currently renting.		
(3) Conation			
Conation	1. I feel that renting a house is a goal that I must strive for at some point in my life.		Merriam-Webster, 1993; Stylos et al., 2016
	2. I feel that the most suitable option for me is to live in the house I am currently renting.		
(4) Attitude			
Attitude	Attitude toward a behavior	1. I feel that I can benefit from a tenancy.	Fishbein and Ajzen, 1975; Taylor and Todd, 1995
		2. I feel that the experience of renting a house is joyful.	
		3. I feel that the quality of my rented house is satisfactory.	
	Attitude toward a target object	1. I feel the house I am currently renting can increase my self-value.	
(5) Tenant satisfaction			
Tenant satisfaction	Service contents	1. I am satisfied with the overall service of the landlord.	Onibokun, 1974; Satsangi and Kearns, 1992; Dimitriades, 2006
		2. I feel satisfied that the landlord is willing to help solve my problems.	
	Service pricing	1. I feel satisfied that the landlord does not increase my rent arbitrarily.	
	Service quality	1. I feel satisfied that the facilities are well-equipped at my rented house.	
	Customer complaints	1. I am satisfied with my landlord’s feelings and the importance they attach to my rights.	
		2. I am satisfied with my landlord’s ability to redress my complaints in a swift manner.	
(6) Tenant loyalty			
Tenant loyalty	Attitudinal loyalty	1. I am willing to renew my tenancy as long as the landlord does not increase my rent arbitrarily.	Oliver, 1997; Hsiao et al., 2006; Chen and Shiu, 2016
		2. I will recommend my landlord’s houses to other potential tenants.	
	Behavioral loyalty	1. I am willing to renew my tenancy when it expires soon.	
(7) Perceived behavioral control			
Perceived behavioral control	Self-efficacy	1. I am capable of acquiring and understanding tenancy-related information.	Ajzen, 1991; Taylor and Todd, 1995
		2. I am capable of renting a house myself.	
		3. I have full control over whether I want to rent a house.	
	Perceived convenience	1. The house I am currently renting is within close proximity to my workplace.	Liu and Wu, 2007
(8) Situational influences			
Situational influences	Passive situations	1. I have a higher willingness to rent a house if I have friends or family living nearby.	Hawkins and Coney, 2003
(9) Subjective norms			
Social norms	Injunctive norms	1. I feel that the tenancy-related opinions of others affect my evaluation of my rented house.	Cialdini and Goldstein, 2004
	Personal norm	1. I feel that there is no need to buy a house in life.	
	Norm of reciprocity	1. I am willing to maintain the environment around my rented house because the landlord is a kind person.	

### Sampling Method and Data Collection

The sample size must be considered before sampling because it affects the accuracy of the estimation results. Assuming a tolerable error of 0.05, a level of significance of 10% (a confidence interval of 90%), the required sample size is 271. Tenants, with the exception of student tenants, who were residing (at the time of study) in eight administrative districts in Kaohsiung City (including Sanmin, Lingya, Cianjin, etc.) were recruited *via* convenience sampling. The researchers administered a questionnaire to each participant face-to-face. The questionnaire survey period lasted from 1 June to 30 June 2019. A total of 315 questionnaires were administered, all of which were recovered. After omitting 15 invalid responses, there were 300 valid responses, indicating an effective recovery rate of 95.2%, which exceeds the required 271 responses for observation purposes.

## Descriptive Statistics of the Sample

### Basic Participant Data

The questionnaire survey results are shown in Appendix 1. Regarding the gender of the survey respondents, male tenants accounted for 46% (139 people) of the sample whereas female tenants accounted for 54% (161 people). Tenants aged between 21 and 30 years accounted for 50% (151 people), followed by those aged between 31 and 40 years, who accounted for 28% (85 people). A majority (68%, 204 people) of the tenants were single, whereas 30% (89 people) were married. Regarding education level, 16% (49 people) of the sample held a postgraduate degree, 60% (179 people) held an undergraduate degree (including 2- and 4-year technical degrees). Regarding occupation, tenants working in the business sector accounted for 28% (83 people), followed by those working in other sectors (26%, 78 people). In terms of length of tenancy, a majority (47%, 140 people) of the sample had been renting their house for 1–3 years, followed by those who had been renting for less than a year (27%, 80 people). In terms of situational influences, 82.3% (247 people) had a friend or family living nearby, whereas 17.7% (53 people) did not.

We divided the 300 valid responses into two equal groups based on the code number. Each group was analyzed for non-response bias *via* the method recommended by Armstrong and Overton (1977). The basic data of the participants were cross-compared in terms of gender, age, marital status, education level, occupation, and length of tenancy. The results showed that the sample data of the recovered questionnaires were consistent with the population structure and had no significant differences, suggesting that there are no significant differences and no overt response bias.

### Reliability and Validity Analysis

#### Reliability Analysis

A reliability analysis, represented by the reliability of measurement variable, is an important indicator of a questionnaires’ stability and consistency. The value of the suitable reliability coefficient should range between 0 and 1 (Chiu, 2006). Reliability of measurement variable, see Table 2.

**TABLE 2:** Analysis of the reliability, factor loading, and average variance extracted of the scale.

Variable	Factor loading (standardized)	Error variance	Reliability of measurement variable	Composite reliability (CR)	Average variance extracted (AVE)	*R*^2^ of structural equation model
Cognition				0.596	0.432	–
Perceived services	0.532	0.717	0.283			
Perceived risk	0.762**	0.419	0.580			
Affection				0.781	0.543	–
Affection 1	0.745**	0.445	0.555			
Affection 2	0.732**	0.464	0.536			
Affection 3	0.734	0.461	0.539			
Conation				0.704	0.543	–
Conation 1	0.712**	0.493	0.507			
Conation 2	0.761	0.421	0.579			
Attitude				0.914	0.843	0.530
Attitude toward a behavior	0.884	0.219	0.905			
Attitude toward a target object	0.951**	0.096	0.781			
Tenant satisfaction				0.877	0.646	0.420
Service contents	0.875**	0.234	0.766			
Service pricing	0.600**	0.640	0.360			
Product quality	0.786**	0.382	0.617			
Customer complaints	0.916	0.161	0.839			
Tenant loyalty				0.881	0.788	0.528
Attitudinal loyalty	0.927**	0.141	0.859			
Behavioral loyalty	0.846	0.284	0.716			
Perceived behavioral control				0.739	0.587	–
Self-efficacy	0.735	0.460	0.634			
Perceived convenience	0.796**	0.366	0.540			
Subjective norms				1.000	1.000	–
Norm of reciprocity	0.759	0				

** indicates p < 0.01.

#### Validity Analysis

In addition to its reliability, the validity of a questionnaire must also be considered. There are three types of validity commonly discussed in research. The first is content validity. The questionnaire in this study centered on the loyalty of tenants. We designed our questionnaire items by referring to previous tenancy-related questionnaires, discussed the contents with expert scholars, and subsequently revised the items and grammar. Therefore, the content validity of our questionnaire should be acceptable and reliable.

Convergent validity is based on the factor loading of each item in each construct. According to Hair et al. (2006), the standardized factor loading of each construct should exceed 0.5, and the greater the average variance extracted (AVE), the greater the reliability of the latent variable in a construct, which collectively indicates good convergent validity. Subjective Norm includes three measurement variables: Injunctive norms, Personal norm, and Norm of reciprocity. The loadings of Injunctive norms and Personal norm are quite low, so that the CR and AVE values are lower than standard levels. We also tried to estimate after removing one of the measurement variables, and the results did not improve anything. We then attempted a factor analysis of these three measured variables and found only one significant factor, and showed that the Norm of reciprocity loading was quite high. Due to this facts, we use Norm of reciprocity to measure Subjective Norm construct as a single variable within the same model for estimation. As shown in Table 2, the standardized factor loadings of all variables, with the exception of personal norms, were greater than 0.5, thus demonstrating that the questionnaire has good convergent validity. Average Variance Extracted (AVE) is higher than 0.5 but we can accept 0.4. Because Fornell and Larcker (1981) said that if AVE is less than 0.5, but composite reliability is higher than 0.6, the convergent validity of the construct is still adequate. Cognition’s AVE value is 0.432, while its CR value is 0.596.

Regarding discriminant validity, Wu and Lin (2002) suggested that it is measured based on the results of a correlation analysis of two concepts. If the two concepts are highly correlated, then both concepts are said to have discriminant validity. In addition, discriminant validity can be determined by examining whether the square root of the AVE of two constructs is greater than the correlation coefficient between the constructs. As shown in Tables 2, 3, the discriminant validity of all construct s were within an acceptable range.

**TABLE 3:** Correlation matrix of the latent variables.

	Cognition	Affection	Conation	Attitude	Tenant satisfaction	Tenant loyalty	Perceived behavioral control	Subjective norms
Cognition	0.657							
Affection	0.001	0.737						
Conation	0.001	0.001	0.737					
Attitude	0.257	0.314	0.605	0.918				
Tenant satisfaction	0.167	0.203	0.392	0.648	0.804			
Tenant loyalty	0.419	0.112	0.217	0.443	0.457	0.887		
Perceived behavioral control	0.001	0.001	0.001	0.001	0.001	0.141	0.766	
Subjective norms	0.629	0.001	0.001	0.162	0.105	0.577	0.001	1.000

*The diagonals represent the square root of the AVE of each construct.*

## Empirical Results and Analysis

The empirical results of this study are presented in terms of the fit of the conceptual framework and the estimation results of the linear structural model.

### Fit of the Conceptual Framework

Bagozzi and Yi (1988) proposed three criteria for assessing the fit of a conceptual framework: overall model fit, preliminary fit criteria, and the fit of a model’s internal structure. We used all three criteria to measure the fit of our model.

#### Preliminary Fit Criteria

Bagozzi and Yi (1988) proposed five preliminary fit criteria for a model: 1. Measurement errors must not be negative; 2. Measurement errors must achieve a level of significance level; 3. The relevant absolute value of estimated parameters must not be too close to 1; 4. The factor loadings must not be too low (lower than 0.5) or too high (higher than 0.95); 5. The standard errors must not be too high. As shown in Table 2, the factor loadings of the measurement items of the eight latent variables all achieved a level of significance and ranged from 0.595 to 0.918 m, except for personal forms. The *R*^2^ of the three structural equations in this study were 0.590, 0.448, and 0.578. In short, the preliminary fit criteria of this study were within an acceptable range.

#### Fit of the Model’s Internal Structure

The fit of a model’s internal structure mainly assesses the level of significance of the estimation parameters as well as the reliability of each indicator and latent variable. Bagozzi and Yi (1988) proposed three indicators of the fit of a model’s internal structure: 1. The individual item reliability, where 0.50 is the threshold for determining the statistical significance of each factor loading. As shown in Table 2, with the exception of personal norms (0.285), all the other factor loadings were greater than 0.50 and were statistically significant. 2. The composite reliability (CR) of a latent variable represents the internal consistency of a construct. According to Fornell and Larcker (1981), a CR greater than 0.60 indicates high reliability, which in turn is indicative of the high consistency of an indicator. With the exception of cognition (0.596), the CR of all other variables was greater than 0.6. 3. The AVE of a latent variable reflects the explanatory power of the latent variable on the variance of each measured variable. A high AVE indicates that the latent variable has a high reliability and convergent validity. Fornell and Larcker (Fornell and Larcker, 1981) concluded that an AVE greater than 0.5 indicates good reliability. According to Table 2, with the exception of cognition (0.432), the AVE of all other variables was greater than 0.50 and were within an acceptable range. To summarize, the fit of our model’s internal structure was acceptable.

#### Overall Model Fit

The overall fit of the model is mainly used to assess how fit the overall model is for the data. Bagozzi and Yi (1988) stressed that the fit of a model cannot be determined through a single criterion or indicator. Instead, the test results of the overall model must be considered. According to Hair et al. (1999), there are three types of overall model fit measures: absolute fit measures, incremental fit measures, and parsimonious fit measures, described as follows:

(1) Absolute fit measures

As shown in Table 4, the chi-square statistic in this study was 578.024 (*p* = 0.001) and statistically significant. This means that the model easily rejects the null hypothesis and the conceptual model fits poorly with the sample data structure. In other words, the conceptual framework differs from the observed data structure. Because the chi-square statistic is easy affected by the sample size, whereby a large sample size will increase the chi-square statistic and therefore reject the null hypothesis (accept the alternative hypothesis), it is necessary to consider other fit indices for evaluating the fit of the overall model (Chiu, 2006). Hair et al. (1999) recommended that a goodness of fit index (GFI), comparative fit index (CFI), and normed fit index (NFI) larger than 0.90 and a root mean square residual (RMR) smaller than 0.05 are indicative of an acceptable model fit. According to Yu (2006), a root mean square error of approximation (RMSEA) smaller than 0.05 indicates an excellent fit, whereas an RMSEA smaller than 0.8 indicates a reasonable fit. In our study, the normal chi-square statistic (χ^2^/*df*) was 4.817, the GFI was 0.801, the RMR was 0.197, and the RMSEA was 0.113, which were all close to an acceptable fit.

**TABLE 4:** Fit indicators of the conceptual framework.

Test statistic		Benchmark for ideal fit	Results
Absolute fit measures	*x^2^* (*P*-value)	578.024	0.001
	χ^2^/*df*	Smaller than 5	4.817
	*GFI*	Greater than 0.90	0.801
	RMR	Favorably smaller	0.197
	*RMSEA*	Favorably smaller, ideal if smaller than 0.05	0.113
Incremental fit measures	*AGFI*	Greater than 0.90	0.717
	*NFI*	Greater than 0.90	0.831
	*CFI*	Greater than 0.90	0.860
Parsimonious fit measures	*PNFI*	Greater than 0.50	0.652
	_*PCFI*_	Greater than 0.50	0.674
			

(2) Incremental fit measures

Incremental fit measures assess the improvement in the fit by comparing a preset model and an independent model. As shown in Table 4, the incremental fit measures AGFI, NFI, and CFI were 0.717, 0.831, and 0.860, respectively, which were all approximately close to an acceptable range.

(3) Parsimonious fit measures

Parsimonious fit measures the fit that each parameter is able to obtain. As shown in Table 4, the parsimonious fit measures PNFI and PCFI were 0.652 and 0.674, respectively, and were all within an acceptable range. Based on all the aforementioned values, the overall fit of the conceptual framework was acceptable.

### Structure Equation Modeling Estimation Results and Discussion

**FIGURE 2:**
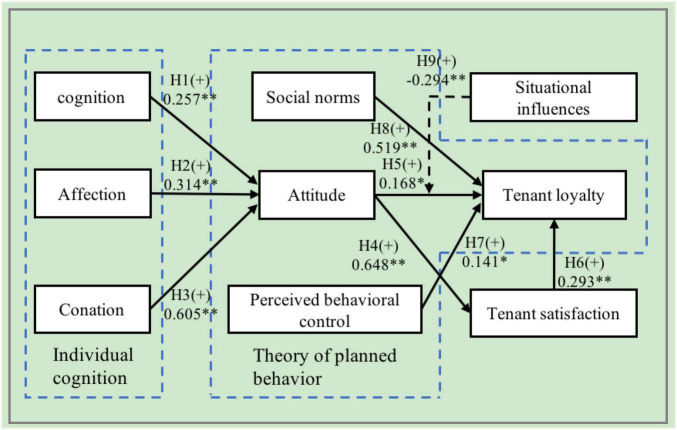
The theoretical structural equation model with standardized coefficients.

As shown in Table 5 and Figure 2, the empirical results derived in this study were explained through an SEM with standardized coefficients. The estimated coefficient of the influence of cognition on attitude was 0.257 and attained a 1% level of significance. Therefore, cognition has a positive and significant influence on attitude. The higher the level of tenants’ cognition, the better their attitude toward their tenancy. Hypothesis 1 is supported. As shown in a study conducted by Lin et al. (2020) on the correlation among cognition, attitude, career expectation and employment intention with 269 interns from teaching hospitals, the cognition and attitude have a significant relationship with employment intention, while cognition has a significant positive effect on attitude. This result is consistent with that of this study. The estimated coefficient of the influence of affection on attitude was 0.314 and attained a 1% level of significance. Therefore, affection has a positive and significant influence on attitude. Hypothesis 2 is supported.

**TABLE 5:** Estimation results of linear structural equation modeling.

Hypothesis	Inter-variable relationship	Estimated coefficient	Standard error		*t*-value		*p*-value
H1	Cognition → Attitude	0.257	0.121		3.900		0.001**
H2	Affection → Attitude	0.314	0.054		5.385		0.001**
H3	Conation → Attitude	0.605	0.061		8.024		0.001**
H4	Attitude → Tenant satisfaction	0.648	0.065		11.856		0.001**
H5	Attitude → Tenant loyalty	0.168	0.085		2.319		0.020*
H6	Tenant satisfaction → Tenant loyalty	0.293	0.072		4.069		0.001**
H7	Perceived behavioral control → Tenant loyalty	0.141	0.082		2.120		0.034*
H8	Subjective norms → Tenant loyalty	0.519	0.126		4.735		0.001**

**Moderating effects**		**Chi-square (χ^2^)**		***p*-value**		**Hypothesis**	
		**Original model**	**Competing model**				
H9 situational influences × Attitude → Tenant loyalty		843.120	855.853	0.002**		Supported	

* indicates p < 0.05, **indicates p < 0.01.

The estimated coefficient of the influence of conation on attitude was 0.605 and attained a 1% level of significance. Therefore, conation has a positive and significant influence on attitude, and the higher the level of tenants’ conation, the better their attitude toward their tenancy. Hypothesis 3 is supported. The empirical results supported Dick and Basu’s (1994) belief that customer loyalty is regarded as the strength of the relationship between individual relative attitude and repurchase, and customer conation has a positive influence on attitude, which is consistent with the results of this study.

The estimated coefficient of the influence of attitude on tenant satisfaction was 0.648 and attained a 1% level of significance. Therefore, attitude has a positive and significant influence on tenant satisfaction, and the better the tenants’ attitude toward their rented house, the more satisfied they are with their tenancy. Hypothesis 4 is thus supported. This is consistent with Jiboye’s (2008) opinion that attitude is the basis of influencing one’s satisfaction with housing, and with the result of this study that attitude has a positive influence on satisfaction. The estimated coefficient of the influence of attitude on tenant loyalty was 0.168 and attained a 5% level of significance. Therefore, attitude has a positive and significant influence on tenant loyalty. Hypothesis 5 is thus supported. The estimated coefficient of the influence of tenant satisfaction on tenant loyalty was 0.293 and attained a 1% level of significance. Therefore, tenant satisfaction has a positive and significant influence on tenant loyalty. The more satisfied tenants are with their tenancy, the more loyal they are. The literature has shown that customer satisfaction affects their repurchase behaviors (Turel and Serenko, 2006). Tenants who perceive a stronger motivation toward their rented house and have more satisfactory experiences will be more loyal toward their tenancy. Hypothesis 6 is thus supported. This is consistent with the research results of Lee et al. (2021) on house tenancy. That is, when the tenants are satisfied with the rented house, they will continue to rent the house, and the tenant loyalty will be greatly improved.

The estimated coefficient of the influence of perceived behavioral control on tenant loyalty was 0.141 and attained a 5% level of significance. Therefore, perceived behavioral control has a positive and significant influence on tenant loyalty; that is, tenants’ needs, desires, and exchanged values have significant influences on their loyalty. According to Gasiorowska (2014), in product buying and selling, customers are affected by the perceived behavioral control of whether they are able to afford a product. As affordability is a variable that affects purchase intentions, perceived behavioral control will effectively affect purchase intentions. Therefore, tenants who perceive a stronger behavioral control over their rented house are more loyal toward their tenancy. Hypothesis 7 is thus supported. The estimated coefficient of the influence of subjective norms on tenant loyalty was 0.519 and attained a 1% level of significance. Therefore, subjective norms have a positive and significant influence on tenant loyalty. Subjective norms that are shaped by common beliefs extensively affect the behaviors of an individual or a group under specific circumstances. Based on this reasoning, a tenant is affected by subjective norms when society displays a good attitude or appraisal toward the tenancy, and hence the tenant is willing to renew the tenancy. Hypothesis 8 is thus supported. The empirical results support all the hypotheses except for Hypothesis 2 as the estimated coefficient of the influence of affection on attitude failed to attain a level of significance. Interestingly, regarding the degree of influence of perceived behavioral control and subjective norms on tenant loyalty, the results showed that the influence of subjective norms on tenant loyalty was higher than that of perceived behavioral control.

Table 5 presents the results of the analysis of the moderating effects. The original model, also known as the baseline model, indicates no invariance assumptions between cross-samples. The model combination consists of two independent and unrelated groups which are structurally similar with the inclusion of the situational influences-based moderator variable of the availability of friends and family living nearby, the baseline model has a χ^2^ of 843.120 (*df* = 316, *p* < 0.001). The competing model is derived by adding the baseline model into a constraint, whereby the two groups with the situational influences-based moderator variable of the availability of friends and family living nearby have the same path coefficient. The competing model has a χ^2^of 855.853 (*df* = 318, *p* < 0.001). The difference in the chi-square statistic (Δχ^2^) between the original and competing models was 12.733 and was statistically significant. Because the difference between these two models is that the competing model includes a constraint, and the difference in the chi-square statistic is significant, the constraint is invalid, and the path estimates of the situational influences-based moderator variable in both groups are different in terms of attitude (0.447) and loyalty (0.741), as shown in Table 6. Regarding the relationship between attitude and tenant loyalty, the inclusion of the moderator variable of the availability of friends and family living nearby significantly diminished the influence of attitude on tenant loyalty. Situational influences are factors that arise from the factors considered by a customer, as well as the time and place, which subsequently affect the customer’s decision-making process (Hawkins and Coney, 2003). Situational factors can affect customer behaviors at any stage during the purchasing process, and may also shorten, prolong, or halt the purchasing process (Pride et al., 2008).

**TABLE 6:** Path analysis of the moderator variable.

Path	Moderator variable	Estimation of having a friend/family member living nearby	Estimation of not having a friend/family member living nearby	Increase/reduction of coefficient
Attitude →Tenant loyalty	Situation influences	0.447	0.741	−0.294**

** indicates p < 0.01. The situational influences-based variable is the availability of friends and family living nearby.

## Conclusion and Suggestions

### Theoretical Implication

This study focused on tenants who are currently renting a house, seeking to examine the influence of personal variables such as cognition, affection, conation, and attitude, as well as subjective norms, perceived behavioral control, and tenant satisfaction, on tenant loyalty, with situational influences as a moderating variable. SEM was used for analysis. GFI test results show that the conceptual model designed in this study had an acceptable fit, which indicates that the model can be supported. The cognition and affection has a significant effect on the attitude, the same is conation on attitude, attitude on tenant royalty, tenant satisfaction on tenant loyalty, perceived behavior control on tenant royalty, subjective norms on tenant loyalty.

### Practical Implication

Tenants’ cognition and conation have a positive influence on their attitude toward their tenancy. Tenants’ personal cognition and conation toward the public facilities and security of their neighborhood, as well as the services provided by their landlord, enabled the tenants to perceive the benefits of their rented house, improve their self-value, and thereby increase their perception toward their own attitude as a tenant.

As tenants improve their attitude toward their tenancy, they also enhance their self-value and the benefits incurred. Landlords can increase tenants’ satisfaction and loyalty by increasing the quality of their services and incorporating the principles of sustainability into these services. Tenants who are satisfied with their tenancy are more likely to renew their tenancy and recommend their landlord to other potential tenants.

Perceived behavioral control has a positive influence on tenant loyalty. Tenants who are capable of renting a house as well as acquiring tenancy-related information increase their tenant loyalty. This is because they must first comprehend whether they have the need to rent a house; if they have and decide to do so, they are less likely to move away once they understand the information about their tenancy. Subjective norms have a positive influence on tenant loyalty. Subjective norms mostly include the opinions of others toward a rented house as well as whether tenants themselves perceive whether they have the need to buy a house during their lifetime. Society opinions toward a rented house will affect tenants’ loyalty. Regarding the degree of influence of perceived behavioral control and subjective norms on tenant loyalty, the results showed that the influence of subjective norms on tenant loyalty was higher than that of perceived behavioral control. Concerning the relationship between attitude on tenant loyalty, the availability of friends and family living nearby significantly diminishes the influence of attitude on tenant loyalty.

### Suggestions

In the housing rental market, there are two actors: the supplier and the demander. As the present study focused on the demander (tenant), future studies can focus on the supplier (landlord) to elucidate and broaden the depth of information regarding housing rentals. Given that this study did not consider the effects of government subsidies on tenant loyalty, future studies can examine this in more detail, in addition to examining whether policies such as rent rebates and subsidies affect tenant loyalty.

## Data Availability Statement

The raw data supporting the conclusions of this article will be made available by the authors, without undue reservation.

## Author Contributions

CL: concept, method, and writing. WY: method and revised. HC and ZY: revised and response. ZT: data collection. All authors contributed to the article and approved the submitted version.

## Conflict of Interest

The authors declare that the research was conducted in the absence of any commercial or financial relationships that could be construed as a potential conflict of interest.

## Publisher’s Note

All claims expressed in this article are solely those of the authors and do not necessarily represent those of their affiliated organizations, or those of the publisher, the editors and the reviewers. Any product that may be evaluated in this article, or claim that may be made by its manufacturer, is not guaranteed or endorsed by the publisher.

## Appendix

**TABLE A1:** Basic data descriptive statistics.

Variable		Number of respondents	Percentage (%)
Gender0	Male	139	46
	Female	161	54
Age	20 years and below	2	1
	21–30 years	151	50
	31–40 years	85	28
	41–50 years	43	14
	50 years and above	19	6
Marital status	Single	204	68
	Married	89	30
	Other	7	2
Education level	Vocational/high school diploma or below	40	13
	Specialized education	32	11
	University (including 2- and 4-year technical degree)	179	60
	Master’s degree	42	14
	Doctorate degree	7	2
Occupation	Businessperson	83	28
	Industrial	36	12
	Military personnel	7	2
	Public servant	45	15
	Educator	38	13
	Agricultural	2	1
	Homemaker	11	4
	Student	0	0
	Other	78	26
Length of tenancy	Less than 1 year	80	27
	1–3 years	140	47
	4–6 years	45	15
	7–9 years	21	7
	10 years and above	14	5
Availability of friends and family living nearby	Yes	247	82
	No	53	18
